# Compounding a High-Permittivity Thermoplastic Material and Its Applicability in Manufacturing of Microwave Photonic Crystals

**DOI:** 10.3390/ma15072492

**Published:** 2022-03-28

**Authors:** Gerardo Andres Mazzei Capote, Maria Camila Montoya-Ospina, Zijie Liu, Michael Sabatini Mattei, Boyuan Liu, Aidan P. Delgado, Zongfu Yu, Randall H. Goldsmith, Tim Andreas Osswald

**Affiliations:** 1 Department of Mechanical Engineering, University of Wisconsin-Madison, Madison, WI 53706, USA; camila.montoya@wisc.edu (M.C.M.-O.); zliu892@wisc.edu (Z.L.); tosswald@wisc.edu (T.A.O.); 2 Department of Chemistry, University of Wisconsin-Madison, Madison, WI 53706, USA; mattei2@wisc.edu (M.S.M.); aidan_delgado@berkeley.edu (A.P.D.); rhg@chem.wisc.edu (R.H.G.); 3 Department of Electrical and Computer Engineering, University of Wisconsin-Madison, Madison, WI 53706, USA; bliu259@wisc.edu (B.L.); zyu54@wisc.edu (Z.Y.)

**Keywords:** additive manufacturing, material extrusion, compounding, topological structures, twin-screw extrusion

## Abstract

Additive Manufacturing (AM) techniques allow the production of complex geometries unattainable through other traditional technologies. This advantage lends itself well to rapidly iterating and improving upon the design of microwave photonic crystals, which are structures with intricate, repeating features. The issue tackled by this work involves compounding a high-permittivity material that can be used to produce 3D microwave photonic structures using polymer extrusion-based AM techniques. This material was acrylonitrile butadiene styrene (ABS)-based and used barium titanate (BaTiO_3_) ceramic as the high-permittivity component of the composite and involved the use of a surfactant and a plasticizer to facilitate processing. Initial small amounts of the material were compounded using an internal batch mixer and studied using polymer thermal analysis techniques, such as thermogravimetric analysis, rheometry, and differential scanning calorimetry to determine the proper processing conditions. The production of the material was then scaled up using a twin-screw extruder system, producing homogeneous pellets. Finally, the thermoplastic composite was used with a screw-based, material extrusion additive manufacturing technique to produce a slab for measuring the relative permittivity of the material, as well as a preliminary 3D photonic crystal. The real part of the permittivity was measured to be 12.85 (loss tangent = 0.046) in the range of 10 to 12 GHz, representing the highest permittivity ever demonstrated for a thermoplastic AM composite at microwave frequencies.

## 1. Introduction

Additive Manufacturing (AM) techniques have risen to the forefront of engineering given their capacity of reproducing intricate design features that would otherwise be difficult to attain through traditional subtractive manufacturing processes. Additionally, the compressed part production cycle that comes attached to all AM technologies allows for rapid iteration and optimization of part design. These advantages over traditional manufacturing are already being leveraged by certain industrial sectors, such as aerospace, to reduce the number of parts required for an assembly or increase the efficiency of processes by exploiting the capability of AM processes to reproduce complex geometric features. However, these advantages also lend themselves well to applications in the fields of photonics and telecommunication systems, particularly in applications where the optimization of geometry is paramount to the success of the finished part [1,2,3,4,5,6,7]. In this realm, the production of microwave photonic devices represents an ideal challenge that can be solved through the use of AM.

The application of AM to microwave photonics has been leveraged to produce simple device designs such as microwave lenses and waveguides, as well as complex geometries of microwave photonic crystals and metamaterials [1,2,3,4,5,6,7]. For these more complex device geometries, and especially for 3D photonic crystals (PCs), the need for high-permittivity materials to realize the desired photonic properties is an outstanding challenge [3,8,9]. Solutions involving AM exist, requiring the use of customized material solutions to deliver the dielectric properties necessary for proper PC performance. Examples include the use of a ceramic suspension in photosensitive resin with the stereolithography process [10,11,12], and the production of composite thermoplastic filaments to be used in material extrusion processes [13].

This research paper explores the compounding of a high-permittivity, thermoplastic material through twin-screw extrusion, and the subsequent manufacture of a preliminary PC structure using a hybrid AM machine that can extrude material in pellet form, as well as support material supplied as a thermoplastic filament. Thermal and rheological characterization was conducted in order to find the optimal processing conditions of the composite in the twin-screw extrusion and in the hybrid AM machine. The relationship between thermal properties and dielectric properties was not investigated in this paper. The composition of this material is based on a recipe published by Wu, Isakov, and Grant [13], and similar findings describing the effect of BaTiO_3_ filler on dielectric properties of composite films [14]. This work represents a case-study for the implementation of pellet-based AM techniques in the manufacture of microwave photonic devices with complex 3D geometries and high-permittivity materials too brittle for filament-based methods. Access to more brittle materials allows the use of higher filler loadings in our composite, yielding the highest-permittivity thermoplastic-based AM composite demonstrated to date for GHz frequencies [3,15,16,17,18]. The utility of the composite is then demonstrated for producing microwave photonic devices by printing and characterizing a 2D photonic crystal and prototyping a 3D photonic structure.

## 2. Materials and Methods

### 2.1. Materials

The ABS/BaTiO_3_/dibutyl phthalate/octyl gallate composite (ABSc) was compounded using ABS as the polymer matrix, BaTiO_3_ as the ceramic filler, dibutyl phthalate as the plasticizer, and octyl gallate as the surfactant. The concentration of the ABSc was as follows: 75% by weight of BaTiO_3_ (nominally 35% by volume, estimated from the density)_,_ 5% by weight of dibutyl phthalate, and 1% by weight of octyl gallate. BaTiO_3_ with 99% purity and <3-micron particle size, dibutyl phthalate, and octyl gallate were purchased from Sigma-Aldrich (Burlington, VT, USA). The concentration of BaTiO_3_ was chosen to be 75% by weight to achieve a higher permittivity than similar formulations previously reported in the literature [13,19].

The ABS used was SABIC CYCOLAC™ MG94 (Riyadh, Saudi Arabia). This material has a reported Melt Flow Index (MFI) of 11.7 g/10 min under test conditions of 230 °C/3.8 kg based on the ASTM D1238 standard [20], where 3.8 kg corresponds to the standard load applied and 230 °C corresponds to the die temperature. The material flow measured in a 10 min period was 11.7 g. ABS-SABIC CYCOLAC™ MG94 has a measured glass transition temperature of 103 °C.

### 2.2. Rheological Characterization

#### 2.2.1. Internal Batch Mixing

The ABSc was prepared in small batches using a C.W. Brabender 3-Piece mixing bowl attached to an Intelli Plasti-Corder Torque Rheometer (Duisburg, Germany), as a preliminary step to characterize the requirements of the compounding process. Mixing occurred at 190 °C and 50 RPM using roller blades with a chamber volume of 60 cm^3^ and a fill factor of 0.7. After mixing, the material was compressed into discs of 25 mm in diameter and 2 mm in thickness using a CARVER Auto Series NE press (Wabash, IN, USA) under 0.5 tons at 190 °C for 1 min. These discs were used in the parallel-plate rheometer to characterize the rheological behavior of the material.

#### 2.2.2. Parallel-Plate Rheometer

Rheology measurements were performed to investigate the properties of ABS and ABSc in the melt. These measurements were made on a TA Instruments AR 2000ex rheometer (New Castle, DE, USA). A 25-mm-diameter parallel steel plate fixture was used to test the materials with a gap of 1.8 mm. Strain sweeps were performed at a constant frequency of 1 rad/s and strains from 0.01% to 100%. The strain sweeps were used to determine the linear viscoelastic region to be explored during small-amplitude oscillatory shear (SAOS) measurements. Frequency sweeps were then performed at 160 °C at a strain of 0.1% and a frequency range of 0.1–100 rad/s.

### 2.3. Thermal Analysis

To understand the thermal stability and proper processing conditions of ABSc, thermogravimetric and calorimetric tests were conducted on the material. Thermogravimetric analysis was conducted using a NETZSCH TG 209 F1 Libra (Selb, Germany) with a resolution of 0.1 µg and a temperature precision of 0.2 °C. Dynamic experiments were performed in an oxygen atmosphere. Samples were ramped from 25 to 600.0 °C at a heating rate of 10 °C/min. Calorimetry experiments were performed with a NETZSCH DSC 214 Polyma (Selb, Germany) with a temperature precision of 0.2 °C. The samples were ramped from 20 to 150 °C at a heating rate of 10 °C/min under nitrogen. These tests were performed to establish the effect of the plasticizer and surfactant upon the glass transition temperature of the material.

### 2.4. Processing

#### 2.4.1. Compounding

After the raw materials (ABS, BaTiO_3_, dibutyl phthalate, and octyl gallate) were pre-processed in an acetone solution, dried, and ground into a coarse powder using a blender, a twin-screw extruder was used to produce the feedstock for AM in the form of ABS composite pellets. The equipment consisted of a Leistritz ZSE 27 twin-screw extruder (Nurnberg, Germany) with a 27 mm screw diameter, an L/D ratio of 36D, and a die with a circular diameter of 3 mm, followed by a water bath destined to cool the extrudate, and a pelletizer. A Schenck process Mechatron Coin-Flex Screw Feeder (Darmstadt, Germany) allowed precise control of the material supply to the throat of the twin-screw extruder. This setup was selected due to the enhanced mixing capabilities of the twin-screw system over a traditional single-screw setup, rooted in higher overall shear forces that allow for deagglomerating the particles of the filler [21].

#### 2.4.2. Single-Screw-Based System Additive Manufacturing

**Figure 1:**
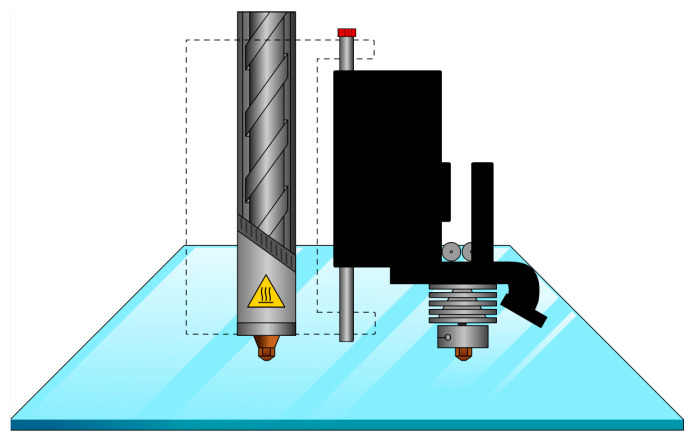
Schematic of single-screw extruder 3D printer.

A Fused Form–FF600+ single-screw extruder 3D printer (Bogotá, Colombia) was employed to produce 3D PCs. This system allows the extrusion of materials that are difficult to supply in filament form [22]. This printer is able to extrude both pellets and filament from two separate nozzles. Pellets in the hopper can be transported to the throat of the printing head using a vacuum, where the pellets are melted and extruded through the heated single screw. With the moving of the printing head, the molten materials are deposited on the print bed in a layer-by-layer manner [23]. In order to avoid interference caused by humidity, the pellets were dried at 60 °C for 4 hours before being poured into the hopper. A schematic of the printer can be seen in Figure 1 where the heat shield and the outer shell of the barrel have been exposed to show the screw.

The general manufacturing process of the samples was as follows: The models generated through CAD software were converted into machine instructions using a slicing engine, and subsequently printed atop an aluminum sheet. This substrate was used to facilitate sample removal post-printing, as well as enhance signal detection in the wave propagation experimental setup. The toolpath files were created using the Simplify3D engine (Cincinnati, OH, USA).

### 2.5. Permittivity Characterization

**Figure 2:**
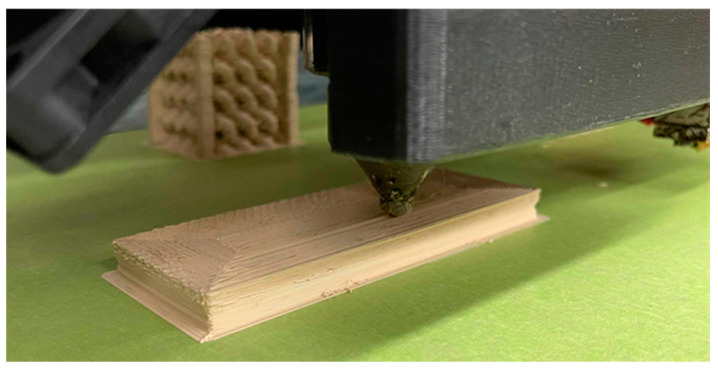
Rectangular slab produced to characterize the permittivity of the material.

The first printed sample, shown in Figure 2, was a rectangular slab for the permittivity characterization with the dimensions of 100 mm × 40 mm × 9 mm. This sample was tested as a Fabry–Parot (FP) cavity in a parallel-plate waveguide. Two antennas fixed on the top plate were linked to a network analyzer, measuring transmission along the waveguide. The slab was placed in the center of the two antennas. Perpendicular to the wave propagation direction is the microwave absorption foam. The antenna will excite the transverse electromagnetic field in the parallel-plate waveguide, which behaves in the same way as a plane wave. It will transmit through the sample slab (40 mm distance) and be received by another antenna.

### 2.6. Scanning Electron Microscopy

Scanning electron microscopy (SEM) was performed on a Hitachi S3400N Variable-Pressure SEM (Tokyo, Japan) with a backscattered electron detector. To ensure that the composite samples (which are insulating) were not damaged by the electron beam during imaging, the chamber pressure was set to 30 Pa, with a probe current of 78 µA, and an accelerating voltage of 11 kV.

### 2.7. Density of ABSc 

The density of four 3D-printed ABSc rectangular slabs (25 mm × 10 mm × 5 mm) was measured to experimentally determine the content of BaTiO_3_. An OHAUS Explorer^TM^ Semi-Micro scale model EX125 (Parsippany, NJ, USA) with 0.01 mg precisions was used in combination with the OHAUS density determination kit (item number: 80253384). This kit uses the Archimedes principle to determine the density of solids. The slab was initially weighed in air and then weighed in distilled water. The scale calculated the density of the slab from the two weights as follows:

(1) $$\rho =\frac{A}{A-B}\;\left(\rho_{0}-\rho_{L}\right)\;+\;\rho_{L}\;$$

where ρ is the density of the sample, *A* is the weight of the sample in air, *B* is the weight of the sample in distilled water, $\rho_{0}$ is the density of the distilled water (0.9982 g/cm^3^ at 20 °C), and $\rho_{L}$ is the density of air (0.0012 g/cm^3^).

### 2.8. Photonic Crystal Simulations

Transmission through the 2D photonic crystal was calculated using a high-speed finite-difference time-domain (FDTD) package for Python 3 (Tidy3D, Flexcompute, Inc.; Cambridge, MA, USA). The size of the simulation region was selected to match the XY dimensions of the experimental parallel-plate waveguide transmission apparatus as closely as possible. The Z dimension of the simulation region was 3 mesh cells tall, with the top and bottom mesh cells filled with a perfect electrical conductor (PEC) to simulate the top and bottom plates of the parallel-plate apparatus. The X and Y boundary conditions were set to the built-in “absorber” settings. Because the PEC keeps the wave confined in the XY plane, the choice of Z boundary conditions is not critical. Periodic boundary conditions were chosen instead of absorbers to reduce simulation time. A line source and time monitor were placed at positions corresponding to the experimental antenna placement. The X and Y mesh size was set to 32 mesh cells per unit cell, and the simulation time was 25 nanoseconds. Spectra were calculated by taking the Fourier transform of the time-domain signal at the monitor and subtracting the simulated transmission without the crystal present from that of the crystal [24].

The band structure of the 3D photonic crystal was simulated using the MIT Photonic Band Gap package (MPB) for Python 3 (Cambridge, MA, USA) [25]. The simulation treats the crystal as an infinite square lattice using Bloch boundary conditions and calculates the eigenfrequencies for a series of wavevectors. The resolution of the simulation was 32 mesh cells per unit cell, and 16 wavevectors were interpolated between each high symmetry point in the Brillouin zone.

## 3. Results and Discussion

### 3.1. Rheological Properties

#### 3.1.1. Internal Batch Mixing

**Figure 3:**
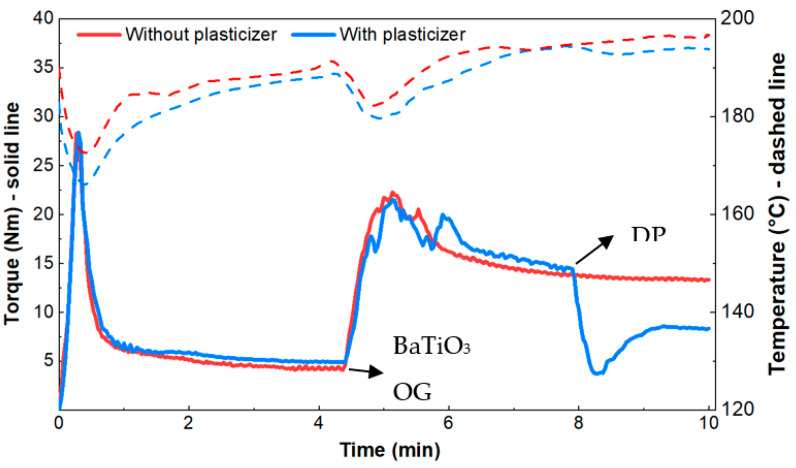
Internal mixing results comparing torque requirements of composites with and without plasticizer where the solid line is torque and dashed line is temperature (50 rpm, 190 °C).

Two batches were prepared to study the effect of the plasticizer on the rheological properties of the composite. One batch contained the plasticizer, and the other did not. The mixing was performed in a multi-step protocol that proceeded as follows: (1) ABS pellets with a measured glass transition temperature of 103 °C were added to the mixer; (2) after approximately 4.5 min, the ABS pellets melted and then the BaTiO_3_ and octyl gallate (OG) were added to the ABS melt and mixed for an additional 3.5 min; lastly, (3) the plasticizer, dibutyl phthalate(DP), was added and mixed for 2 additional minutes. These steps are labeled in Figure 3. BaTiO_3_ and octyl gallate were in powder form, while dibutyl phthalate was in a liquid state. The addition of plasticizer was omitted in the second batch.

The effects that the ceramic filler (BaTiO_3_) and the plasticizer have on the viscosity of the blend were determined by the torque curve shown in Figure 3. The torque, which is related to the viscosity, increases by a factor of approximately four when BaTiO_3_ and OG are added to the ABS melt. The torque recorded at the 5-minute mark was 22 N·m. As BaTiO_3_ is dispersed in the polymer matrix, the torque decreases, approaching a plateau value of 14 N·m, at which point the plasticizer (DP) is fed. The torque decreased from 14 to 8 N·m in this step. This information provides a valuable guide for the torque requirements for the next two processing steps: Compounding in a twin screw and extrusion-based 3D printing.

#### 3.1.2. Parallel-Plate Rheometer

**Figure 4:**
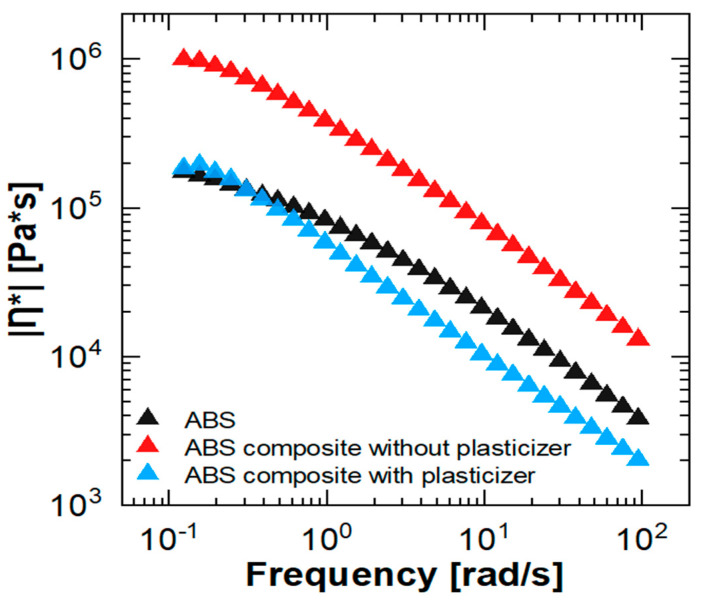
Complex viscosity (|η*|) of ABS and ABSc without and with a plasticizer (0.1% strain, 160 °C).

At 100 rad/s, the measured viscosities (|η*|) for ABS, ABSc without a plasticizer, and ABSc with a plasticizer were 3734, 12,750, and 1953 rad/s, respectively (Figure 4). The high viscosity values recorded in the composite blend without the plasticizer are due to the high content of ceramic filler (36.3 ± 0 volume percent, see below). However, by adding a plasticizer to the composite, the viscosity was decreased by a factor of 6.5. In the frequency range of 0.5–100 rad/s, the viscosity of the ABSc with a plasticizer was even lower than the neat ABS. For this reason, the former blend was pursued for the compounding step and 3D printing.

### 3.2. Thermal Properties

**Figure 5:**
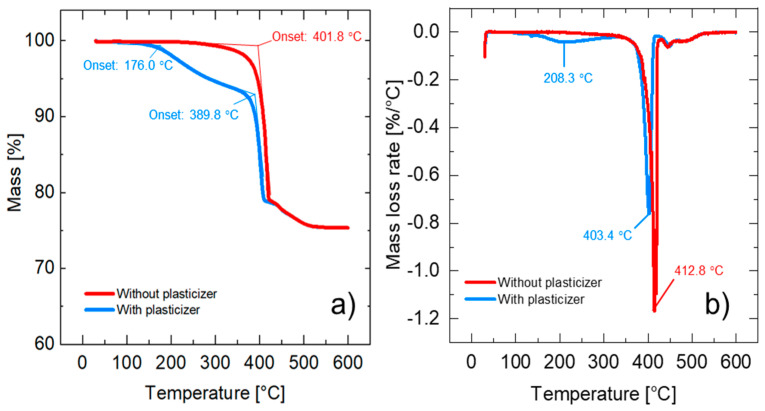
TGA measurements of ABSc with and without plasticizer (10 °C/min, Oxygen): (**a**) Mass loss [%] vs. temperatures [°C]; (**b**) mass loss rate [%/°C] vs. temperature [°C].

Figure 5a shows the TGA results from the composite prepared in the internal batch mixer. ABSc without a plasticizer has a degradation onset temperature of 401.8 °C, which is attributed to the ABS matrix. ABSc with a plasticizer shows two degradation mechanisms. The first degradation onset temperature occurs at 176.0 °C and the second one at 389.8 °C. The mass loss at 176.0 °C is most likely due to the evaporation of the plasticizer, while the mass loss at 389.8 °C corresponds to the degradation of ABS. For this reason, the temperature profiles selected in the twin-screw extruder and in the 3D printer were set to values below 176.0 °C. The first derivative of the mass change with respect to temperature is shown in Figure 5b. Lastly, the residual mass percentage measured at 600.0 °C was 75.3483%, corresponding to BaTiO_3_.

**Figure 6:**
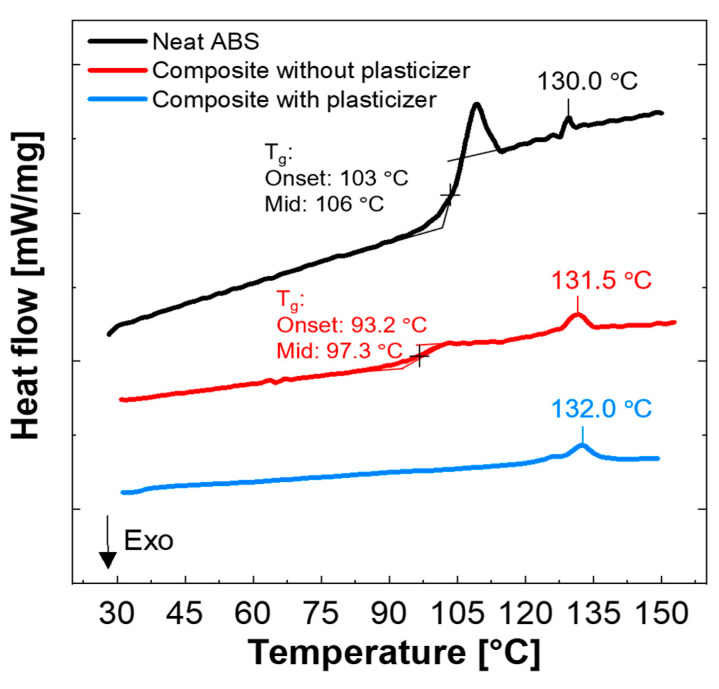
DSC heating curves of neat ABS and ABSc without and with plasticizer (10 °C/min, nitrogen).

The glass transition temperature (*T_g_*) measured in the neat ABS and in ABSc with and without a plasticizer is shown in Figure 6. The neat ABS has an onset *T_g_* of 103 °C with a midpoint of 106 °C corresponding to the styrene component. Moreover, the glass transition of ABS shows a clear relaxation peak. Enthalpy relaxation is a response of amorphous materials annealed below its glass-transition temperature as previously demonstrated by X. Quan et al. [26,27]. The addition of the plasticizer had a significant effect on the glass-transition temperature of ABSc. The *T_g_* of the ABSc with the plasticizer was not detected in the temperature range tested (25–150 °C). Due to limitations in the equipment, the temperature could not be further decreased. Notice that the addition of the surfactant (octyl gallate) also decreased the *T_g_* of the composite by approximately 10 °C. This result aligned with the data reported by Wu et al. [13]. Melting points at 130.0, 131.5, and 132.0 °C were observed in the neat ABS and the ABSc without and with a plasticizer, respectively. This value is consistent with the peak temperature of the acrylonitrile component. The rheological characterization in the parallel-plate rheometer was performed at 160.0 °C. This temperature was selected after analyzing the TGA and DSC results.

### 3.3. Processing Parameters

#### 3.3.1. Compounding Parameters

The extruder speed and the feeding rate were selected to maintain the melt pressure under 500 psi. The extruder speed was set at 50 RPMs, and the material was supplied using a feed rate of 2 kg/h. The optimal temperature profile of the extruder was selected in accordance with the results of the internal batch mixing and thermal characterization of the composite. The temperature profile is listed in Table 1. Note how the temperature is kept below 176 °C to prevent volatilization of the components, as shown in the TGA results.

#### 3.3.2. Additive Manufacturing Parameters

The printing speed was selected by considering both the printing efficiency and sample quality. It is worth noting that the matching of the pellet’s melting speed and the printing speed plays an important role in the integrity of the printed objects and the wear of the screw. The relevant slicing parameters are shown in Table 2. Note that the print temperature chosen was 160 °C, as opposed to 130 °C used in the twin-screw process. Any temperature below this point would prove difficult to print, as the motor that drives the motion of the screw would seize.

### 3.4. Permittivity Characterization

**Figure 7:**
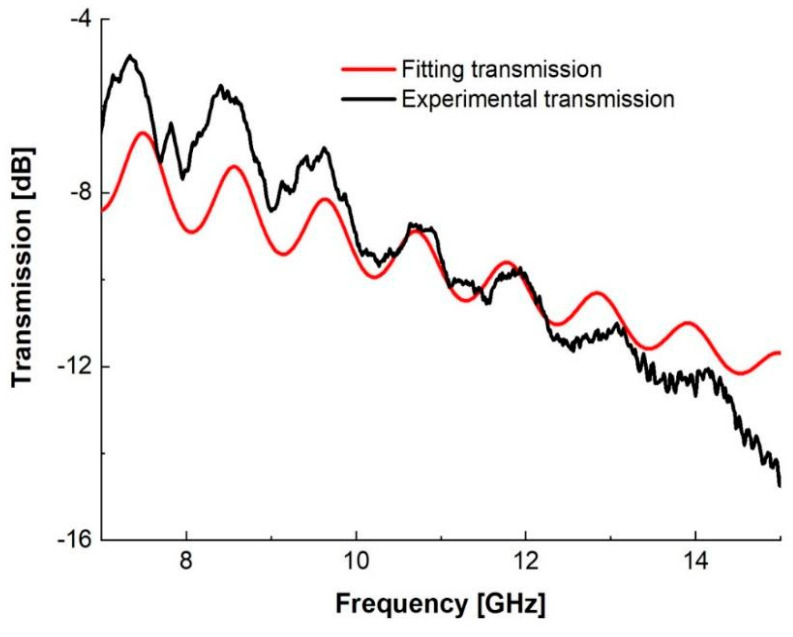
The experimental transmission and theoretical fitting transmission spectrum.

The permittivity was estimated by fitting the theoretical model to the experimental transmission spectrum in Figure 7. The model can be calculated from the multi-beam interference theory [25]. Transmission *T* can be calculated as:

(2) $$T=|\frac{4n_{1}n_{2}}{{\left(n_{1}+n_{2}\right)}^{2}}\frac{e^{ikd}}{1-{\left(\frac{n_{2}-n_{1}}{n_{1}+n_{2}}\right)}^{2}e^{i2kd}}{|}^{2}$$

where $n_{1},n_{2}$ are the refractive indexes of air and the slab, respectively, and k represents the wave vector $k=n_{2}\frac{2\pi f}{c}$, where f is the frequency, c is the speed of light, and d is the thickness of the sample. This differs from the expression in reference [28] by a “phase factor” $e^{ikd}$ in the numerator since there is absorption in our slab, represented by the imaginary part of $n_{2}$. Adding the slab thickness into the theoretical transmission expression, *T* can be fitted to the experiment transmission spectrum by adjusting the real and imaginary parts of the permittivity of the slab. The fitting (Figure 7) shows that the permittivity is $\epsilon_{2}=n_{2}^{2}=12.85+0.59i$ from 10 to 12 GHz. The mismatch between the experimental result and the theoretical curve, observed between 7 and 10 GHz and after 12 GHz, is due to the dispersion of the material (frequency dependence of the permittivity). The real part of permittivity is the highest demonstrated to date for thermoplastic AM materials at GHz frequencies due to increased BaTiO_3_ content enabled by printing with a screw extrusion system rather than filament [3,15,16,17,18]. The loss tangent is 0.046, slightly higher than those reported in Reference [3] (0.027) and Reference [13] (~0.03), as expected, due to our increased BaTiO_3_ loading [3,13]. The fitting is based on minimizing the mean square error of two transmissions at 7 to 15 GHz. The mean absolute percentage error is 11.8% at 7 to 15 GHz and 2.7% at 10 to 12 GHz.

### 3.5. Characterization of ABSc Microstructure

**Figure 8:**
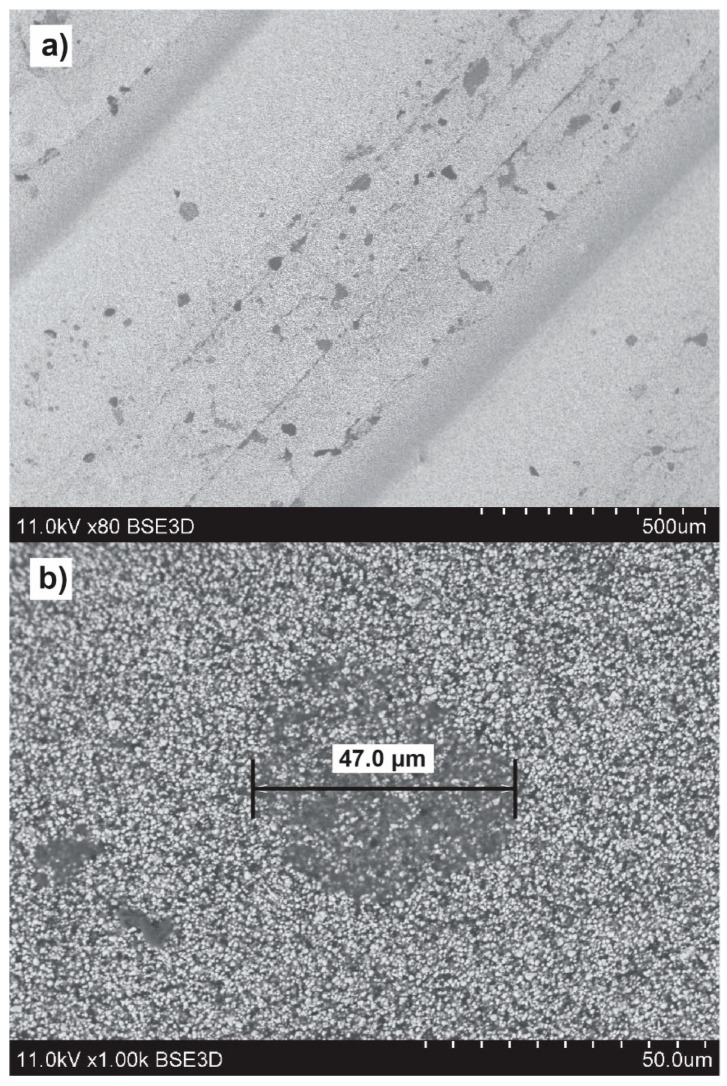
Backscattered electron micrographs of the surface of a 3D-printed layer of ABSc at 80× (**a**) and 1000× (**b**) magnification showing large areas of isotropically distributed barium titanate particles (white dots). Small (<50 µm) voids are visible on the surface. At 1000× magnification, individual BaTiO_3_ particles are visible and are less densely distributed inside the void.

Variable-pressure scanning electron microscopy was used to visualize the microstructure of ABSc on the surface of a 3D-printed sample (Figure 8). The images show large continuous areas of isotropically distributed barium titanate particles (white dots), with some small (<50 µm) voids throughout (darker regions). Such voids have been observed previously in filaments of similarly formulated composites, but the microstructure of the voids has not been investigated [13]. The voids can influence the permittivity of parts printed from our ABSc. However, the scale of the voids (<50 µm) is several orders of magnitude smaller than the wavelengths at which we have characterized the permittivity (~2 cm at 15 GHz). The measured permittivity is therefore a volume average of the permittivity of the composite and the air present inside the voids, yielding a slightly lower effective permittivity than that expected for the ABSc in the absence of voids. These air voids could be formed by ceramic particles clustering and preventing the resin (ABS) from flowing inside these gaps during the compounding process.

### 3.6. BaTiO_3_ Content Determination

The presence of voids in ABSc yields a measured permittivity that is slightly lower than that of the same ABSc formulation without voids. It is thus important to ask what the final BaTiO_3_ content is in the printed ABSc samples. This was determined from the experimentally measured BaTiO_3_ weight percent (75.3483 ± 0.0007%, Section 2.3 and Section 3.2) and the measured density of the printed composite (2.90 ± 0.03 g/cm^3^, Section 2.7). This was calculated as:

(3) $$\%vol_{BaTiO_{3}}\;=\;\frac{\left(\rho_{ABSc}\right)\left(\%wt_{BaTiO_{3}}\right)}{\rho_{BaTiO_{3}}}$$

where $\%vol_{BaTiO_{3}}$ is the percent by volume of the ceramic in the ABSc, $\rho_{ABSc}$ is the measured density of ABSc, $\rho_{BaTiO_{3}}$ is the density of BaTiO^3^ (6.02 g/cm^3^), and $\%wt_{BaTiO_{3}}$ is the percent by weight of ceramic in ABSc. The resulting calculated percent by volume of BaTiO3 in the ABSc is 36.6 ± 0.4%, consistent with the higher permittivity of the ABSc compared to a similar formulation in Reference [13].

### 3.7. Crystal Characterization

**Figure 9:**
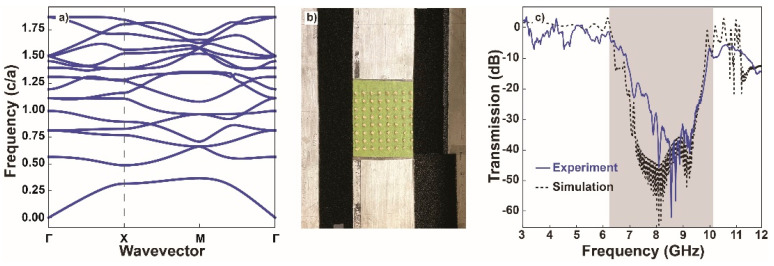
(**a**) Simulated band structure for a square lattice of dielectric rods. (**b**) Parallel-plate waveguide apparatus containing a 2D photonic crystal. (**c**) The measured and simulated (finite-difference time-domain) transmission through the crystal show excellent agreement with a ~45 dB decrease in transmission inside the photonic band gap (highlighted in grey). The direction along which the transmission was measured corresponds to the dashed line in (**a**).

Following characterization of the permittivity and BaTiO_3_ content, its applicability to producing 3D-printed photonic materials was demonstrated. First, a simple 2D lattice consisting of an 8 × 8 square array of dielectric rods with a lattice constant of 1.5 cm, a rod diameter of 3.6 mm, and a rod height of 7.35 mm was examined. The simulated band structure is shown in Figure 9a. Using the same parallel-plate waveguide apparatus as for the permittivity measurements, the transmission through the crystal along the lattice vector was measured. A photograph of the crystal inside the apparatus is shown in Figure 9b. The experimentally measured and simulated transmissions through the crystal along the lattice vector are plotted in Figure 9c and show excellent agreement for the position and depth of the band gap (8.6 GHz and −50 dB, respectively). The width of the band gap is expected to increase as the permittivity increases. A large gap–midgap ratio (the ratio of the gap width to the gap center frequency) for the band gap of our 2D crystal due to the high permittivity of ABSc was expected [29]. Indeed, a ~3 GHz-wide band gap was observed, corresponding to a large gap–midgap ratio of 0.35.

**Figure 10:**
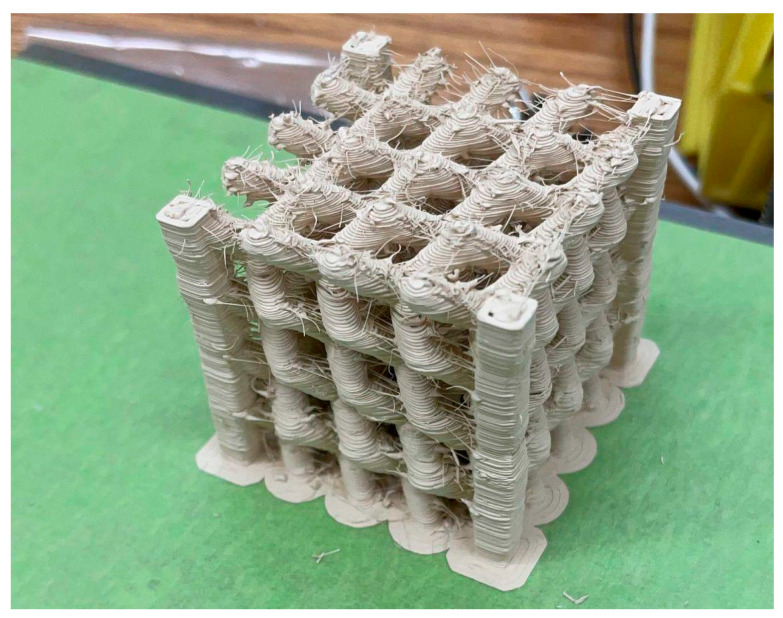
3D photonic crystal.

Next, a prototypical 3D photonic crystal was produced, inspired by the topological photonic crystal proposed by Yang, et al. [24]. Initial attempts to reproduce the geometry resulted in poor fidelity to the original CAD file. The pillars in the structure were difficult to reproduce without the use of support structures in the four corners. The file was then modified to include built-in support, and this structure is displayed in Figure 10. The erratic strands that can be seen on the surface of the structure are the result of the inability to completely pause the dripping of materials in the screw printer due to residual pressure within the barrel during relocating, non-depositing movements of the gantry.

**Figure 11:**
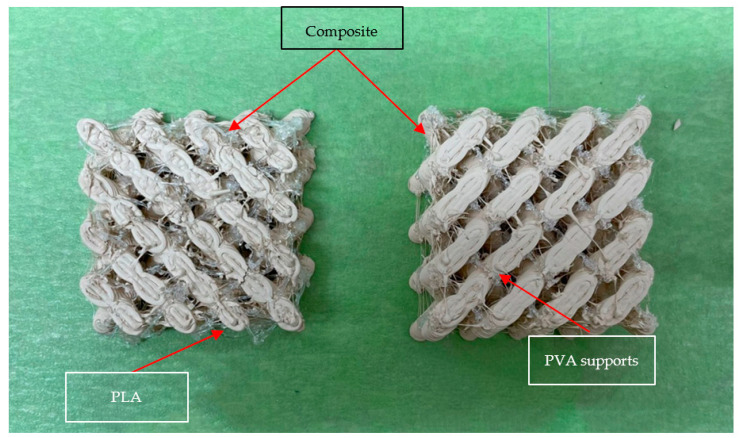
Partial print with PLA support structures (**left**) and PVA (**right**).

Additional attempts were made using the original CAD, and support structures printed using PVA (Polyvinyl Alcohol) and PLA (Polylactic Acid) (Matterhackers; Lake Forest, CA, USA) yielded undesirable results. Both PVA and PLA had poor adhesion to the surface of ABSc, resulting in poor surface quality and failed support structure generation. An example can be seen in Figure 11. This image shows prints stopped prior to completion to illustrate the poor adhesion between the support material and the composite.

**Figure 12:**
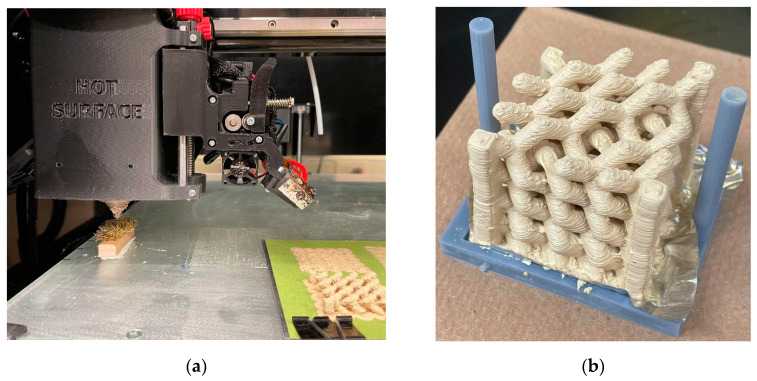
(**a**) Nozzle cleaning system on the printing bed; (**b**) 3D photonic crystal with surface defects removed by immersing in acetone for 30 min.

Based on the poor surface quality observed, the use of secondary print material for support was discarded, and two major changes were introduced to the manufacturing process to ensure higher fidelity to the original STL geometry. The first is a self-cleaning motion trajectory added during the printing process, where the print head would travel at the beginning of each layer to a fixed position that contained a brass brush, allowing the nozzle to be cleaned of unwanted debris and print droll in a consistent manner. This protocol can be seen in Figure 12a. Secondly, the printed crystals are post-treated in an acetone bath, where the object was placed on a small holder lined with aluminum foil, via digital light processing using Anycubic Basic Grey resin (Shenzhen, China). The holder was then immersed in a covered container of acetone for 30 min with no agitation. The finished part is shown in Figure 12b where the vastly improved surface finish can be appreciated when compared to Figure 10.

## 4. Conclusions

The methodology of producing a small batch of the material using an internal batch mixer and experimenting on it prior to scaling up production allowed the researchers to fine-tune the processing window while minimizing trial and error. The addition of a plasticizer and surfactant required the material to be processed at 130 °C in the twin-screw process, producing homogeneous pellets of material. This temperature had to be adapted to 160 °C for the single-screw extrusion printing due to the limitations of the machine. At this temperature, the viscosity of the material is comparable to the viscosity of neat ABS. In contrast, the material produced without a plasticizer had complex viscosity an entire order of magnitude larger.

Producing a slab of ABSc through 3D printing allowed for measuring the complex permittivity of the material using a parallel-plate microwave waveguide. The real part of the permittivity was measured to be 12.85 in the frequency range of 10 to 12 GHz, with a loss tangent of 0.046—the highest microwave permittivity reported to date for a thermoplastic AM composite. We then demonstrated the utility of our ABSc in the production of microwave photonic devices by printing a 2D photonic crystal and experimentally verifying its expected transmission behavior. A prototypical 3D photonic crystal was also produced. The complex geometry of the 3D crystal required the addition of support structures. The prints produced using support produced with polylactic acid (PLA) and polyvinyl alcohol (PVA) proved unsuccessful, given the poor adhesion between ABSc and the support material. The alternative that yielded the highest fidelity to the original CAD file was the model that included the support pillars on the vertical edges of the structure and was produced using the composite itself. All structures had surface defects, resulting from the impossibility of stopping the flow of material during travel moves due to residual pressure within the barrel of the single-screw extruder. These surface defects can largely be prevented by cleaning the nozzle between each layer during the print. Any remaining surface defects can be removed simply by immersion in acetone for 30 min. The 3D lattice printed in this work is a prototypical design meant to demonstrate the fabrication of complex 3D structures using our ABSce and contains too few unit cells to be a functional photonic crystal. Future work will involve scaling up this design to produce functional photonic crystal devices, experimentally characterizing their band structures.

## Figures and Tables

**Table 1:** List of temperature profiles used in twin-screw extruder.

Zone Section	Zones 1 & 2	Zone 3	Zones 4 to 8	Die
Temperature [°C]	120	125	130	130

**Table 2:** List of relevant slicing parameters.

Parameter	Selected Value
Layer height [mm]	0.25
Path width [mm]	0.8
Extrusion multiplier	1.8
First layer height [%]	100
Print speed [mm/min]	500
Infill [%]	100
Infill orientation	[90° 0°]
Print temperature [°C]	160
Zone 1 temperature [°C]	75
Zone 2 temperature [°C]	130
Bed temperature [°C]	60
Outline perimeters [–]	3

## Data Availability

Not applicable.
